# Job vacancies at the European Centre for Disease Prevention and Control (ECDC)

**DOI:** 10.2807/1560-7917.ES.2021.26.1.2101072

**Published:** 2021-01-07

**Authors:** Eurosurveillance editorial team

**Affiliations:** 1 European Centre for Disease Prevention and Control (ECDC), Stockholm, Sweden

The European Centre for Disease Prevention and Control (ECDC) invites applications for the following positions:

 

**Scientific Officer Mathematical Modelling**

The application deadline expires on 29 January 2021. 

 

**Principal Expert microbial safety of substances of human origin**

The application deadline expires on 1 February 2021. 

 

**Scientific Officer Behaviour Change**

The application deadline expires on 5 February 2021. 

****

For more information, please visit the ECDC website.

